# Spiral ligament fibrocyte-derived MCP-1/CCL2 contributes to inner ear inflammation secondary to nontypeable *H. influenzae*-induced otitis media

**DOI:** 10.1186/1471-2334-10-314

**Published:** 2010-10-28

**Authors:** Jeong-Im Woo, Huiqi Pan, Sejo Oh, David J Lim, Sung K Moon

**Affiliations:** 1 Division of Cell Biology & Genetics, House Ear Institute, 2100 West 3rd Street, Los Angeles, CA, USA; 2 Department of Cell and Neurobiology, University of Southern California, 1975 Zonal Avenue, KAM 500, Los Angeles, CA, USA; 3 Brain and Mind Research Institute, University of Sydney, 94 Mallette St, Camperdow NSW 2050, Australia

## Abstract

**Background:**

Otitis media (OM), one of the most common pediatric infectious diseases, causes inner ear inflammation resulting in vertigo and sensorineural hearing loss. Previously, we showed that spiral ligament fibrocytes (SLFs) recognize OM pathogens and up-regulate chemokines. Here, we aim to determine a key molecule derived from SLFs, contributing to OM-induced inner ear inflammation.

**Methods:**

Live NTHI was injected into the murine middle ear through the tympanic membrane, and histological analysis was performed after harvesting the temporal bones. Migration assays were conducted using the conditioned medium of NTHI-exposed SLFs with and without inhibition of MCP-1/CCL2 and CCR2. qRT-PCR analysis was performed to demonstrate a compensatory up-regulation of alternative genes induced by the targeting of MCP-1/CCL2 or CCR2.

**Results:**

Transtympanic inoculation of live NTHI developed serous and purulent labyrinthitis after clearance of OM. THP-1 cells actively migrated and invaded the extracellular matrix in response to the conditioned medium of NTHI-exposed SLFs. This migratory activity was markedly inhibited by the viral CC chemokine inhibitor and the deficiency of MCP-1/CCL2, indicating that MCP-1/CCL2 is a main attractant of THP-1 cells among the SLF-derived molecules. We further demonstrated that CCR2 deficiency inhibits migration of monocyte-like cells in response to NTHI-induced SLF-derived molecules. Immunolabeling showed an increase in MCP-1/CCL2 expression in the cochlear lateral wall of the NTHI-inoculated group. Contrary to the *in vitro *data, deficiency of MCP-1/CCL2 or CCR2 did not inhibit OM-induced inner ear inflammation *in vivo*. We demonstrated that targeting MCP-1/CCL2 enhances NTHI-induced up-regulation of MCP-2/CCL8 in SLFs and up-regulates the basal expression of CCR2 in the splenocytes. We also found that targeting CCR2 enhances NTHI-induced up-regulation of MCP-1/CCL2 in SLFs.

**Conclusions:**

Taken together, we suggest that NTHI-induced SLF-derived MCP-1/CCL2 is a key molecule contributing to inner ear inflammation through CCR2-mediated recruitment of monocytes. However, deficiency of MCP-1/CCL2 or CCR2 alone was limited to inhibit OM-induced inner ear inflammation due to compensation of alternative genes.

## Background

Otitis media (OM) is one of the most common infectious diseases in children and it is estimated to cost more than $5 billion annually in the U.S. for the management of OM [1,2]. OM is not a life-threatening disease, but causes inner ear complications such as sensorineural hearing loss (SNHL) [3] and vertigo [4]. OM-induced inner ear complications of children are clinically important since even a mild hearing loss can interfere with a child's language development, [5] and balance dysfunction is associated with delays in achievement of motor milestones [6].

The incidence of OM-induced SNHL may be underestimated since OM results in hearing loss in ultrahigh frequencies as well as transient hearing threshold shifts, which cannot be easily detected with a conventional hearing test [7,8]. OM-induced SNHL is believed to be caused by immune-mediated damage due to inner ear inflammation, which is initiated by the entry of bacterial molecules of OM pathogens into the inner ear through the round window membrane [9]. Animal studies of pneumococcal OM demonstrate cochlear damage such as hair cell loss [10] and pathologic changes in the cochlear lateral wall [11,12].

Since pneumococcal conjugate vaccination has caused a shift in the predominant OM pathogens, nontypeable *H. influenzae *(NTHI) is becoming the most commonly-isolated organism of OM [13]. NTHI is a small Gram-negative bacterium, existing as a commensal organism in the human nasopharynx. The clinical course of NTHI-induced OM is less severe and is more chronically associated with middle ear effusion compared to that of pneumococcal OM [14]. Thus, it is expected that NTHI molecules contained in middle ear effusion may gain more chances to enter the inner ear, but it is poorly understood how NTHI-induced OM leads to inner ear inflammation.

Recently, we have demonstrated that spiral ligament fibrocytes (SLFs) release chemokines in response to OM pathogens [15]. Among the inner ear cells, we proposed SLFs as a main responder to the inflammatory stimuli because SLFs, one of the most abundant inner ear cell types, express pathogen recognition receptors such as toll-like receptor 2 (TLR2) [16]. We believe that SLFs recognize OM pathogens entering the inner ear and release chemokines recruiting inflammatory cells such as monocytes. Monocytes are known to infiltrate the cochlea in chronic middle ear inflammation [17] as well as after acoustic trauma [18]. Previously, we demonstrated that SLFs up-regulate monocyte chemoattractant protein-1 (MCP-1/CCL2) in response to NTHI through TLR2-dependent NF-kB activation [16]. Therefore, we hypothesize that OM pathogen-induced SLF-derived MCP-1/CCL2 plays a major role in inner ear inflammation secondary to OM. We here show that NTHI-induced SLF-derived MCP-1/CCL2 contributes to OM-induced inner ear inflammation through the CCR2-mediated recruitment of monocytes. However, we found that inhibition of MCP-1/CCL2 or CCR2 alone is limited to inhibit OM-induced inner ear inflammation since targeting the MCP-1/CCL2 gene affects expression of alternative genes such as MCP-2/CCL8.

## Methods

### Reagents

Recombinant MCP-1/CCL2 and RS102895 (a chemical inhibitor of CCR2) was purchased from Sigma (St. Louis, MO). Recombinant viral CC chemokine inhibitor (CCI) was purchased from R&D Systems (Minneapolis, MN).

### Bacterial culture and preparation of bacterial lysate

NTHI strain 12 that was used is originally a clinical isolate from the middle ear fluid of a child with acute OM [19]. To restore virulence, 250 μl of NTHI suspension (10^5 ^cfu/ml) was inoculated into the murine peritoneal cavity and recovered with peritoneal lavage the next day. The NTHI lysate was prepared as described previously [20]. Briefly, a single colony of NTHI was harvested from a chocolate agar plate, inoculated into 30 ml of brain heart infusion broth supplemented with NAD (3.5 μg/ml) and incubated overnight. The supernatant was discarded after centrifugation at 10,000 × *g *for 10 min. The pellet was resuspended in 10 ml of phosphate-buffered saline (PBS) and sonicated to lyse the bacteria. The lysate was then centrifuged at 10,000 × *g *for 10 min, and the supernatant was collected.

### Cell culture

In this study, we used the rat SLF cell line, immortalized with adenovirus type 12-simian virus 40 hybrid virus [21], primary murine SLFs, THP-1 cells (ATCC, Manassas, VA) and primary murine splenocytes. The rat SLF cell line was maintained in DMEM (Invitrogen, Carlsbad, CA) supplemented with 10% fetal bovine serum, penicillin (100 units/ml), and streptomycin (0.1 mg/ml). Primary SLFs were cultured from explants of the mouse cochlear lateral walls as previously described [16]. THP-1 cells were maintained in suspension in RPMI 1640 containing 10% fetal bovine serum, penicillin (100 U/ml), streptomycin (100 mg/ml) and 2-mercaptoethanol (50 μM) at a density of 5 × 10^5 ^cells/ml. For harvesting splenocytes, mouse spleen was aseptically dissected after euthanization and was homogenized between the frosted ends of the glass slides in the DMEM. The homogenized spleen was passed through the nylon mesh and cells were treated with ACK lysis buffer to remove red blood cells.

### Animals

C57BL/6, MCP-1^-/-^, CCR2^-/- ^and TNF^-/- ^mice were purchased from the Jackson Laboratory (Bar Harbor, ME). All aspects of animal handling were performed according to the approved IACUC guidelines (HE1131-09-03). 10^7 ^cfu of live NTHI was suspended in 10 μl of saline and was transtympanically inoculated into the middle ear of the young adult male mice using a 27 G needle and syringe under the surgical microscope. As a control, sterile normal saline was inoculated with the same procedure. Animals were sacrificed at 3, 5 and 7 days after inoculation and temporal bones were dissected. After fixation and decalcification, the temporal bone was embedded in paraffin and was serially sectioned through the mid-modiolar plane at a thickness of 10 μm. H & E staining and immunolabeling was performed for the histological analysis. According to the classification of human temporal bones with a history of OM [22], we divided these inner ear inflammatory responses into three groups, 1) no inflammation; 2) serous labyrinthitis (showing accumulation of serous substances and hemorrhage with none or minimal infiltration of inflammatory cells); and 3) purulent labyrinthitis (showing massive infiltration of inflammatory cells). For immunolabeling, endogenous peroxidase activity was quenched with 0.3% H_2_O_2, _and nonspecific binding sites were blocked with horse serum (1:500). Sections were incubated with polyclonal rabbit anti-MCP-1 antibody (1:200, Santa Cruz Biotechnology, Santa Cruz, CA) and biotinylated anti-rabbit IgG antibody (Vector Laboratories, Burlingame, CA). Bound secondary antibodies were detected by the reaction of diaminobenzidine tetrahydrochloride with peroxidase that was attached by the avidin-biotin complex method.

### Migration and invasion assays

After overnight starvation, SLFs were exposed to the NTHI lysate (0.1 μg/ml) for 12 h and the conditioned medium was collected. As a control, the conditioned medium of SLFs was separately collected without NTHI exposure. Migration assays were performed using CytoSelect™24-well cell migration assay kit (Cell Biolabs, San Diego, CA) with the polycarbonate membrane inserts (5 μm pores) according to the manufacturer's instructions. Briefly, suspended THP-1 cells or primary splenocytes were added to each insert (300 μl) at a density of 1 × 10^6 ^cell/insert and the conditioned medium was added to the lower chamber. Cells were allowed to migrate for 12 h, and migrated cells were lysed and detected with CyQuant GR dye solution (Invitrogen, Carlsbad, CA). Fluorescence was measured with a plate reader at 480 nm/520 nm. For the invasion assays, polycarbonate membrane inserts (8 μm pores) coated with basement membrane matrix were used.

### Real-time quantitative PCR and silencing of MCP-1/CCL2

Real-time quantitative PCR was performed as described previously [20]. Briefly, after SLFs were exposed to the NTHI lysate, total RNA was extracted using the RNeasy kit (Qiagen, Valencia, CA), and cDNA was synthesized using the TaqMan reverse transcription kit (Applied Biosystems). Real time quantitative PCR was performed with Syber Green Master Mix and primers specific to mouse MCP-1/CCL2 (NM_011333: 5'-TCACCTGCTGCTACTCATTCACCA-3'; 5'-TACAGCTTCTTTGGGACACCTGCT-3'), MCP-2/CCL8 (NM_021443: 5'-AGCCTTGAACCTTCACACCTGAGT-3'; 5'-CCAGGCACCATCTGCTTGTAACAT-3'), MCP-3/CCL7 (NM_013654: 5'-ACCAACCTAGGAGCCAAGAAGCAA-3'; 5'-AAGACCATTCCTTAGGCGTGACCA-3'), CCR1 (NM_009912: 5'-ACCAGTTCCTCAGCAAAGGATGGA-3'; 5'-TAGGACATTGCCCACCACTCCAAT-3') and CCR2 (NM_009915: 5'-TCCTCAGTACCTTTGCAACTGCCT-3'; 5'-AGCAAGACTTCTGTCCCTGCTTCA-3'). The cycle threshold (CT) values were determined according to the manufacturer's instructions, and the relative quantity of mRNA was determined using the 2^-(ΔΔCT) ^method [23]. CT values were normalized to the internal control (GAPDH), and the results were expressed as a fold change of mRNA, taking mRNA levels of the non-treated group as 1. For the silencing of MCP-1/CCL2 expression, cells were transfected with MCP-1-specific siRNA (s128379, Ambion, Austin, TX) using the siPORT™ NeoFX™ transfection agent (Ambion), according to the manufacturer's instruction. As a control, the negative control siRNA (Ambion) was transfected in parallel.

### Statistics

All experiments were carried out in triplicate and repeated two or three times. Results were expressed as means + standard deviations. Statistical analysis was performed using Student's *t *test and Fisher's exact test with significance considered to be a *p *value of < 0.05.

## Results and Discussion

### 1. Inoculation of NTHI into the middle ear leads to labyrinthitis

**Figure 1:**
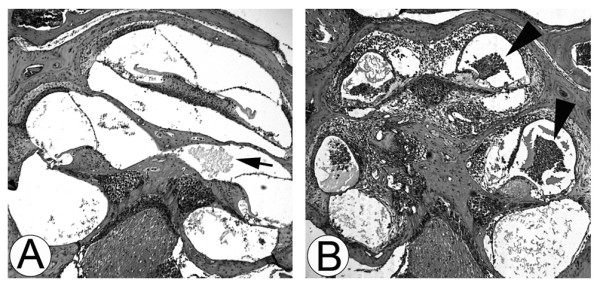
**Inner ear inflammation secondary to NTHI-induced middle ear infection**. Live NTHI was inoculated into the murine middle ear through the tympanic membrane and temporal bones were harvested at 7 days after bacterial inoculation. H & E staining shows that middle ear infection results in serous labyrinthitis (A) and purulent labyrinthitis (B). Note accumulation of serous substances with hemorrhage (arrow) and massive infiltration of inflammatory cells (arrowhead) in the cochlear spaces. Original magnification: ×50.

It is known that middle ear inflammation leads to inner ear inflammation resulting in inner ear dysfunction [24]. To establish a murine model for OM-induced inner ear inflammation, live NTHI was inoculated into the murine middle ear through the tympanic membrane after restoration of virulence by animal peritoneal passing. The pilot study determined 10^7 ^cfu as the optimal bacterial dosage of NTHI for the development of OM-induced inner ear inflammation in C57BL/6 mice. Murine temporal bones were harvested at 7 days after bacterial inoculation and histological analysis was performed. An incubation period shorter than 7 days was insufficient for the development of OM-induced inner ear inflammation. Histological analysis showed that middle ear infection leads to inner ear inflammatory responses such as accumulation of serous substances, hemorrhage and infiltration of inflammatory cells (Figure 1). As shown in Table 1, transtympanic inoculation of live NTHI significantly caused inner ear inflammation in 93% of ears including serous (21%) and purulent (71%) labyrinthitis, compared to 50% in the control animals (*p *< 0.05). It was noted that labyrinthitis remains after clearance of middle ear inflammation, indicating inner ear inflammation occurs as a complication of OM.

**Table 1:** NTHI-induced inner ear inflammation in wild type, MCP-1-deficient and CCR2-deficient mice.

Mice	Inoculation (mice)	No inflammation (ears)	Serous labyrinthitis (ears)	Purulent labyrinthitis (ears)	Fisher's exact test
Wild type	Saline^a^ (4)	4	2	2	
	NTHI^b^ (7)	1	3	10	a vs. b*: p *< 0.05
MCP-1^-/-^	NTHI^c^ (5)	0	4	6	b vs. c: NS
CCR2^-/-^	NTHI^d^ (5)	0	0	10	b vs. d: NS

The incidence of SNHL attributed to OM is known to be less than 5% although it may be underestimated due to difficulties in testing [14,25-27]. In our mouse model, NTHI-induced OM caused labyrinthitis more frequently than expected, which may occur due to a thinner round window membrane in rodents than humans [28]. The round window membrane is the major route from the middle ear to the inner ear, since it is the only non-bony barrier between two structures. Its permeability is known to be affected by exposure to inflammatory conditions [29-31]. Inflammatory mediators or bacterial molecules such as endotoxin or exotoxin compromise the round window membrane permeability barrier and diffuse into the perilymph [9,32,33]. Once in the inner ear, these molecules induce pathologic changes including inflammation of the perilymphatic space and the spiral ligament, strial swelling, sensory cell degeneration [33] and fibrinogen deposition [11].

### 2. NTHI-induced SLF-derived molecules attract THP-1 cells

**Figure 2:**
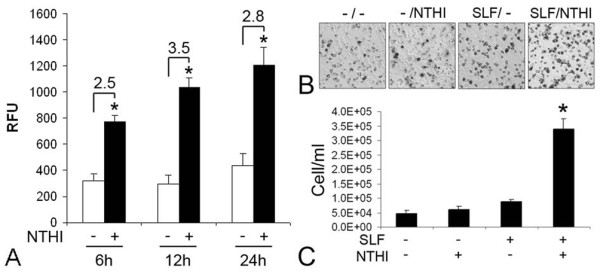
**Attraction of THP-1 cells by SLFs-derived molecules**. The culture medium of SLFs was collected after 6, 12 and 24 h with or without exposure to the NTHI lysate, respectively. Migration assays show that the conditioned medium of NTHI-exposed SLFs highly attracts THP-1 cells compared to that of NTHI-unexposed SLFs (A). RFU: relative fluorescence unit. Invasion assays show that NTHI-induced SLF-derived molecules enhance invasion of THP-1 cells (dark and solid) to the extracellular matrix on the semi-permeable membrane with pores (light and open) (B). It is noted that NTHI lysate per se does not affect migration and invasion of THP-1 cells, compared to the NTHI-induced SLF-derived molecules (C). *: *p *< 0.05. The experiments were performed in triplicate and repeated twice. Values are given as the mean ± standard deviation (n = 3).

After entering the cochlea from the middle ear through the round window membrane, bacterial molecules in the perilymph of the scala tympani are expected to make contact with the cochlear structures such as the spiral ligament laterally and the osseous spiral lamina medially. The spiral ligament is a connective tissue structure made up of SLFs and collagen fibrils that forms the lateral wall of the cochlea. In particular, the spiral ligament-facing scala tympani is not completely covered with lining epithelial cells, thus allowing free flow of perilymph to SLFs [34]. To determine if SLFs release molecules attracting monocytes in response to NTHI, we performed migration assays using THP-1 cells (the human acute monocytic leukemia cell line) and the conditioned medium of NTHI-exposed SLFs. Migration assays showed that THP-1 cells migrated in response to the conditioned medium of NTHI-exposed SLFs more actively than the conditioned medium without NTHI exposure, indicating that SLFs release molecules attracting THP-1 cells in response to NTHI (Figure 2A). Next, we performed invasion assays to determine if NTHI-induced SLF-derived molecules enhance invasion of THP-1 cells. We found that THP-1 cells invaded the extracellular matrix in response to the conditioned medium of NTHI-exposed SLFs (Figure 2B &2C). However, the NTHI molecules per se did not affect migration and invasion of THP-1 cells, compared to NTHI-induced SLF-derived molecules.

We believe that monocytes play a critical role in OM-induced inner ear inflammation, similar to acoustic trauma, which also induces infiltration of monocytes [18,35,36]. Monocytes are a major element of the innate immunity defense to pathogen invasion, but their overly-robust response can lead to pathological sequelae resulting in tissue damage. Monocytes recruited to the cochlea are thought to be involved in both propagation of tissue damage, resulting in sensorineural hearing loss, and local disposal of injured cochlear cells as a wound-healing process.

### 3. MCP-1/CCL2 is a main attractant for THP-1 cells among the NTHI-induced SLF-derived molecules

**Figure 3:**
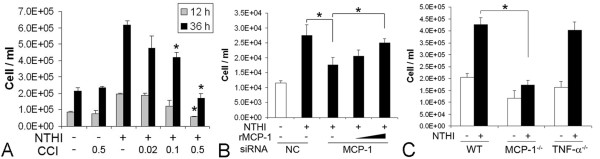
**Identification of the main molecule attracting THP-1 cells among NTHI-induced molecules derived from SLFs**. Viral CC chemokine inhibitor (CCI) blocks migration of THP-1 cells responding to the NTHI-induced SLF-derived molecules (A). Migration assays were performed using THP-1 cells and the conditioned medium of NTHI-exposed SLFs with and without treatment of CCI (0.02, 0.1 and 0.5 μg/ml) for 12 h and 36 h. Silencing of MCP-1/CCL2 inhibits migration of THP-1 cells attracted by the conditioned medium of NTHI-exposed SLFs, which is restored by addition of the recombinant MCP-1/CCL2 (rMCP-1, 0.1 and 1 ng/ml) (B). SLFs were transfected with either non-specific siRNA (NC) or MCP-1-specific siRNA (MCP-1) and culture medium was collected after 12 h with and without exposure to the NTHI lysate. Results were expressed relative to the fold change of mRNA levels, taking the value of the NC siRNA-treated group as 1. THP-1 cell migration in response to the conditioned medium of NTHI-exposed SLFs is inhibited more than 60% by MCP-1/CCL2 deficiency, but not by TNF-α deficiency (C). Primary SLFs were cultured in the lower compartment of the migration chamber and were exposed to the NTHI lysate. THP-1 cells were co-cultured in the upper compartment, and migrated cells to the lower chamber were quantitated using a hemocytometer. The experiments were performed in triplicate and repeated twice. Values are given as the mean ± standard deviation (n = 3). *: *p *< 0.05.

**Figure 4:**
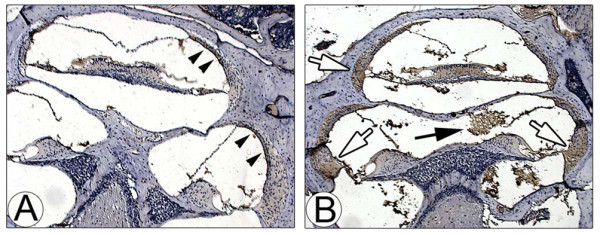
**SLF-derived MCP-1/CCL2 is associated with inner ear inflammation secondary to NTHI-induced OM *in vivo***. Murine temporal bones were harvested at 7 days after transtympanic inoculation of saline (A) and live NTHI (B). Immunolabeling shows MCP-1/CCL2 is highly expressed in the spiral ligaments (white arrows) and spiral limbus as well as the serous substances (black arrow) of the NTHI-inoculated mice. In contrast, it is noted that the cochlea of the saline-inoculated group weakly expresses MCP-1/CCL2 except the stria vascularis (arrowheads). Original magnification: ×50.

Monocytes are known to be attracted by a number of molecules such as complement C5a and monocyte chemotactic proteins (MCPs) [37,38]. To determine a critical molecule involved in attraction of THP-1 cells among the NTHI-induced SLF-derived molecules, we conducted migration assays with a viral CC chemokine inhibitor (vCCI). As shown in Figure 3A, recombinant vCCI suppressed migration of THP-1 cells attracted by the NTHI-induced SLF-derived molecules in a dose-dependent manner, indicating the involvement of CC chemokines. Among CC chemokines, we explored silencing MCP-1/CCL2 expression, based on our previous data showing that SLFs released high levels of MCP-1/CCL2 upon exposure to the OM pathogens [15]. SLFs were transfected with either non-specific siRNA (NC) or rat MCP-1-specific siRNA. Cells were exposed to the NTHI lysate and the conditioned culture medium was harvested. Migration assays demonstrated that THP-1 cells poorly migrated in response to the conditioned medium of MCP-1-silenced SLFs, and this inhibition was restored by the addition of recombinant MCP-1/CCL2 in a dose-dependent manner (Figure 3B). qRT-PCR showed that the MCP-1-specific siRNA inhibits MCP-1/CCL2 expression more than 60% compared to the NC siRNA (data not shown). Next, we sought to determine if MCP-1/CCL2 deficiency affects chemotactic activity of NTHI-induced SLF-derived molecules. We isolated and cultured primary SLFs from the wild-type mice, the MCP-1-deficient mice and the TNF-α-deficient mice as described previously [16]. Migration assays showed that MCP-1 deficiency, not TNF-α deficiency, significantly inhibits THP-1 cell migration attracted by NTHI-induced SLF-derived molecules more than 55% (Figure 3C). Altogether, these findings indicate that MCP-1/CCL2 is a main attractant for THP-1 cells among the NTHI-induced SLF-derived molecules *in vitro*. To determine if MCP-1/CCL2 is associated with inner ear inflammation secondary to NTHI-induced OM *in vivo*, immunolabeling of NTHI-inoculated temporal bone sections was performed using a polyclonal anti-MCP-1 antibody. As shown in Figure 4, spiral ligaments highly expressed MCP-1 in NTHI-inoculated mice, compared to saline-inoculated mice. Particularly, the serous substances in the cochlear spaces were labeled strongly with an anti-MCP-1 antibody, suggesting that they may contain the released MCP-1/CCL2.

MCP-1/CCL2, one of the most studied chemokines, is produced by a variety of cells such as fibroblasts and macrophages, either constitutively or after induction by inflammatory stimuli [39-41]. MCP-1/CCL2 is known to be involved in a number of immune diseases including atherosclerosis and insulin-resistant diabetes [42,43]. In this study, we showed that SLF-derived MCP-1/CCL2 contributes to OM-induced inner ear inflammation through recruiting monocytes. In addition to inflammatory infiltration, monocytes constitutively migrate from blood to tissue for maintaining resident macrophages. Cochlear resident macrophages are found to be localized in the spiral ligament and the spiral ganglion area [44], but further studies are necessary to elucidate a mechanism related to the homing of monocytes to the cochlea.

### 4. CCR2 is required for the migration of THP-1 cells in response to the NTHI-induced SLF-derived molecules

**Figure 5:**
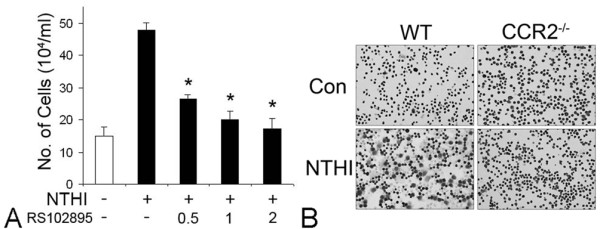
**Requirement of CCR2 for migration of monocytes in response to NTHI-induced SLF-derived molecules**. Migration assays show that the CCR2 inhibitor (RS 102895) markedly inhibits migration of THP-1 cells attracted by NTHI-induced SLF-derived molecules, in a dose-dependent manner (A). Giemsa staining of the migrated splenocytes shows that CCR2 deficiency inhibits migration of monocyte-like cells in response to the NTHI-induced SLF-derived molecules (B).

According to the classification based on the spacing of the N-terminal cysteine residues, CCR2 belongs to the β-subclass of chemokine receptors, which serves as a receptor for the natural ligands such as MCP-1/CCL2 and MCP-2/CCL8 [45]. To determine an involvement of CCR2 in monocyte recruitment induced by SLF-derived molecules, migration assays were performed after blocking of CCR2 with a CCR2 inhibitor (RS 102895). Migration assays showed that RS 102895 markedly inhibits migration of THP-1 cells in response to NTHI-induced SLF-derived molecules in a dose-dependent manner (Figure 5A). There are two isoforms of CCR2 as a result of alternative splicing [46]. CCR2a is the major isoform expressed by vascular smooth muscle cells while monocytes and activated NK cells express predominantly the CCR2b [47]. Compared to CCR2a, CCR2b is more sensitive to MCP-1/CCL2, which is selectively inhibited by RS 102895.

To determine if CCR2 deficiency affects chemotactic activity of NTHI-induced SLF-derived molecules, we performed migration assays using primary splenocytes of mice. Migrated cells were collected and Giemsa staining was performed after cytospin preparation. It was found that NTHI-exposed conditioned medium of SLFs attracts large cells with abundant cytoplasm resembling activated monocytes in the wild type mice (Figure 5B). In contrast, monocyte-like cells of CCR2-deficient splenocytes poorly migrated in response to NTHI-induced SLF-derived molecules, indicating the requirement of CCR2 for the migration of monocyte-like cells in response to NTHI-induced SLF-derived molecules. MCP-1/CCL2 interacts only with CCR2, whereas CCR2 responds to both agonists (e.g., MCP-2/CCL8) and antagonists (e.g., CCL11) [48]. Therefore, it is challenging to understand the *in vivo *implications of interactions between chemokines and corresponding receptors.

### 5. Deficiency of MCP-1/CCL2 or CCR2 alone is limited for the inhibition of OM-induced inner ear inflammation

**Figure 6:**
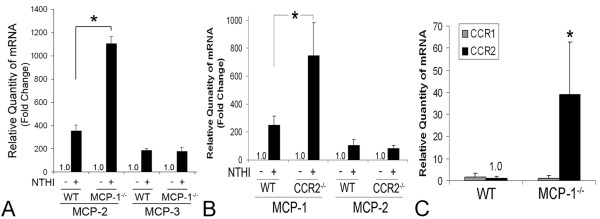
**Deficiency of MCP-1/CCL2 and CCR2 affects NTHI-induced regulation of other CC chemokines**. qRT-PCR shows that NTHI-induced up-regulation of MCP-2/CCL8 is markedly enhanced in SLFs of MCP-1^-/- ^mice compared to the wild type mouse (A). It is noted that the regulation of MCP-3/CCL7 is not affected. CCR2 deficiency enhances NTHI-induced up-regulation of MCP-1/CCL2, but not MCP-2/CCL8 (B). Primary SLFs derived from the wild type, MCP-1-deficient and CCR2-deficient mice were exposed to the NTHI lysate for 3 h. Results were expressed relative to the fold change of mRNA levels, taking the value of the non-treated group as 1. MCP-1/CCL2 deficiency up-regulates CCR2 expression in the splenocytes, but CCR1 expression is not affected (C). mRNA levels of CCR1 and CCR2 were relatively shown, taking the CCR2 level of the wild type splenocytes as 1. *: *p *< 0.05. The experiments were performed in triplicate and repeated three times. Values are given as the mean ± standard deviation.

To determine if the deficiency of MCP-1/CCL2 or CCR2 affects OM-induced inner ear inflammation *in vivo*, live NTHI was transtympanically inoculated into the middle ear of the wild type, MCP-1/CCL2-deficient and CCR2-deficient mice. Contrary to our *in vitro *data, neither MCP-1/CCL2 deficiency nor CCR2 deficiency significantly inhibited OM-induced inner ear inflammation (Table 1). Previously, we have shown that lysozyme P expression is up-regulated in lysozyme M-deficient mice [49], which suggests that the targeting of one gene affects the regulation of its alternative genes for compensation [50]. Moreover, since it has been reported that targeting of the MCP-1/CCL2 gene affects lipopolysaccharide-induced MCP-3/CCL7 production [51], we hypothesized that MCP-1/CCL2 deficiency by the insertion of a neo-gene cassette is compensated by the up-regulation of other MCPs. We compared NTHI-induced transcriptional regulation of MCP-2/CCL8 and MCP-3/CCL7 in the wild type mouse and the MCP-1/CCL2-deficient mouse. As shown in Figure 6A, NTHI-induced up-regulation of MCP-2/CCL8 was highly enhanced by the targeting of the MCP-1/CCL2 gene in SLFs, whereas the regulation of MCP-3/CCL7 was not affected. Considering that MCP-1/CCL2 interacts only with CCR2 but MCP-2/CCL8 activates CCR1 and CCR3 in addition to CCR2, we suggest that SLF-derived MCP-2/CCL8 may induce inner ear inflammation instead of MCP-1/CCL2.

It has been reported that CCR2 deficiency did not suppress noise-induced monocyte migration into the cochlea and dramatically increased noise-induced hair cell death [52], suggesting either an over-compensation of CCR2 deficiency or the presence of a CCR2-mediated protective mechanism. Thus, we investigated the effect of CCR2 deficiency on the regulation of CCR2 ligands and other chemokine receptors. As shown in Figure 6B, CCR2 deficiency enhanced NTHI-induced up-regulation of MCP-1/CCL2 in SLFs in accordance with previous reports [53,54]. In contrast, MCP-2/CCL8 expression did not appear to be affected by CCR2 deficiency. We also found that MCP-1/CCL2 deficiency highly up-regulates the basal expression of CCR2 but CCR1 expression is not affected, taking the CCR2 level of the wild type splenocytes as 1 (Figure 6C). Altogether, we suggest that the deficiency of MCP-1/CCL2 or CCR2 is compensated by up-regulation of alternative genes in the inner ear *in vivo*, resulting in the discrepancy between *in vitro *and *in vivo *data.

In agreement with our findings, acoustic trauma-induced cochlear inflammation is known to be unaffected by single MCP-1/CCL2- or CCR2-deficiency [52]. Moreover, it has been reported that macrophage migration through the blood-brain barrier is not influenced by single MCP-1/CCL2- or CCR2-deficiency, but double-deficient mice lead to a virtual absence of blood-borne macrophage recruitment [55]. Cochlea and brain have in common a blood-tissue barrier [56]. In other systems without a blood-tissue barrier, single MCP-1/CCL2 deficiency is found to effectively inhibit MCP-1/CCL2-mediated diseases such as atherosclerosis [57] and diabetes [58]. Therefore, we suggest that this barrier may be involved in compensation of single MCP-1/CCL2- or CCR2-deficiency, but further studies are necessary.

It remains unclear how targeting an MCP-1/CCL2 gene regulates expression of other MCPs. Recently, it has been reported that lipopolysaccharide-induced MCP-3/CCL7 production is down-regulated by the targeting of an MCP-1/CCL2 gene with the insertion of a neo-gene cassette, but is up-regulated by the deletion of the MCP-1/CCL2 gene [51]. Since the MCP-1/CCL2 gene is located upstream from other MCPs genes, the disruption of the MCP-1/CCL2 gene may alter the DNA conformation at the locus, resulting in aberrant transcription of genes such as MCP-2/CCL8 and MCP-3/CCL7 that are downstream from the MCP-1/CCL2 gene. In addition to MCP-2/CCL8, we believe that other genes, which are not tested in the study, may be affected by the targeting of MCP-1/CCL2 and contribute to the discrepancy of our *in vitro *and *in vivo *findings.

## Conclusions

In this study, we showed that SLFs, in response to NTHI, release molecules attracting monocytes. Among NTHI-induced SLF-derived molecules, we demonstrated that MCP-1/CCL2 is a major attractant enhancing migration and invasion of monocytes. We also found that CCR2 is required for monocyte recruitment responding to NTHI-induced SLF-derived molecules. However, deficiency of MCP-1/CCL2 or CCR2 alone produced limited inhibition of OM-induced inner ear inflammation since targeting a gene affected expression of alternative genes. In summary, we suggest that NTHI-induced SLF-derived MCP-1/CCL2 contributes to OM-induced inner ear inflammation through CCR2-mediated recruitment of monocytes. We believe that our findings will enable us to further understand the molecular pathogenesis of OM-induced inner ear inflammation.

## List of Abbreviations

OM: otitis media; NTHI: nontypeable *Haemophilus influenzae*; SLF: spiral ligament fibrocyte; SNHL: sensorineural hearing loss; MCP: monocyte chemotactic protein; CCL: CC chemokine ligand; CCR: CC chemokine receptor.

## Competing interests

The authors declare that they have no competing interests.

## Authors' contributions

JIW performed most of the experiments and equally contributed to this work with SKM.

HP was involved in histological works and animal husbandry.

SJO was involved in animal dissection and silencing of genes.

DJL, a recipient of DC005025 and DC6276, supervised the histological analysis.

SKM, a recipient of DC 8696, designed this work, analyzed data, and prepared the manuscript.

All authors read and approved the final manuscript.

## Pre-publication history

The pre-publication history for this paper can be accessed here:

http://www.biomedcentral.com/1471-2334/10/314/prepub
